# Publisher’s Note: We Changed Page Numbers to Article Numbers for Articles Published in *Audiology Research* Volume 1–Volume 10, Issue 1

**DOI:** 10.3390/audiolres12020012

**Published:** 2022-02-23

**Authors:** Audiology Research Editorial Office

**Affiliations:** MDPI AG, St. Alban-Anlage 66, 4052 Basel, Switzerland; audiolres@mdpi.com

*Audiology Research* [1] was published by PAGEPress from Volume 1 (2011) to Volume 10, Issue 1 (2020). From Volume 10, Issue 2 (2020), *Audiology Research* has been published by MDPI.

Previous articles in Volumes 1–10, published by PAGEPress in Open Access under a CC-BY (or CC-BY-NC-ND) license, are now hosted by MDPI on mdpi.com as a courtesy and upon agreement with PAGEPress.

To standardize the metadata format of all previous articles, MDPI republished 125 articles in Volume 1–Volume 10, Issue 1, with article numbers replacing page numbers (Table 1).

## Figures and Tables

**Table 1:** MDPI has changed the page numbers to article numbers for 125 articles in Volumes 1–10.

DOI	Previous Page Number	Current Article Number
10.4081/audiores.2011.e1	1	e1
10.4081/audiores.2011.e2	3–5	e2
10.4081/audiores.2011.e3	6–8	e3
10.4081/audiores.2011.e4	9–15	e4
10.4081/audiores.2011.e5	16–18	e5
10.4081/audiores.2011.e6	19–21	e6
10.4081/audiores.2011.e7	22–24	e7
10.4081/audiores.2011.e8	25–27	e8
10.4081/audiores.2011.e9	28–33	e9
10.4081/audiores.2011.e10	34–39	e10
10.4081/audiores.2011.e11	40–41	e11
10.4081/audiores.2011.e12	42–48	e12
10.4081/audiores.2011.e13	49–54	e13
10.4081/audiores.2011.e14	55–57	e14
10.4081/audiores.2011.e15	58–60	e15
10.4081/audiores.2011.e16	61–63	e16
10.4081/audiores.2011.e17	64–68	e17
10.4081/audiores.2011.e18	69–70	e18
10.4081/audiores.2011.e19	71–73	e19
10.4081/audiores.2011.e20	74–78	e20
10.4081/audiores.2011.e21	79–81	e21
10.4081/audiores.2011.e22	82–85	e22
10.4081/audiores.2011.e23	86–87	e23
10.4081/audiores.2011.e24	88–90	e24
10.4081/audiores.2011.e25	91–96	e25
10.4081/audiores.2011.e26	97–110	e26
10.4081/audiores.2011.e27	1–8	e27
10.4081/audiores.2011.e28	9–12	e28
10.4081/audiores.2011.e29	13–17	e29
10.4081/audiores.2011.e30	18–22	e30
10.4081/audiores.2011.e31	23–29	e31
10.4081/audiores.2011.ed	1	ed
10.4081/audiores.2012.e1	1–3	e1
10.4081/audiores.2012.e2	4–7	e2
10.4081/audiores.2012.e3	8–11	e3
10.4081/audiores.2012.e4	12–16	e4
10.4081/audiores.2012.e5	17–24	e5
10.4081/audiores.2012.e6	25–29	e6
10.4081/audiores.2012.e7	30–38	e7
10.4081/audiores.2012.e8	39–42	e8
10.4081/audiores.2012.e9	43–47	e9
10.4081/audiores.2012.e11	57–63	e11
10.4081/audiores.2012.e12	64	e12
10.4081/audiores.2012.e13	65–76	e13
10.4081/audiores.2012.e14	77–85	e14
10.4081/audiores.2012.e15	86–93	e15
10.4081/audiores.2013.e1	1–9	e1
10.4081/audiores.2013.e2	10–15	e2
10.4081/audiores.2013.e3	16–25	e3
10.4081/audiores.2013.e4	26–31	e4
10.4081/audiores.2013.e5	32–41	e5
10.4081/audiores.2013.e6	42–47	e6
10.4081/audiores.2013.e7	48–51	e7
10.4081/audiores.2013.e8	52–56	e8
10.4081/audiores.2013.e9	57–62	e9
10.4081/audiores.2014.80	36–39	80
10.4081/audiores.2014.88	14–20	88
10.4081/audiores.2014.89	9–13	89
10.4081/audiores.2014.91	5–8	91
10.4081/audiores.2014.93	1–4	93
10.4081/audiores.2014.99	56–60	99
10.4081/audiores.2014.101	28–35	101
10.4081/audiores.2014.102	40–45	102
10.4081/audiores.2014.106	21–27	106
10.4081/audiores.2014.108	52–55	108
10.4081/audiores.2014.110	46–51	110
10.4081/audiores.2015.105	1–4	105
10.4081/audiores.2015.111	5–8	111
10.4081/audiores.2015.113	21–29	113
10.4081/audiores.2015.116	44–49	116
10.4081/audiores.2015.117	9–19	117
10.4081/audiores.2015.120	30–33	120
10.4081/audiores.2015.121	65–68	121
10.4081/audiores.2015.128	34–37	128
10.4081/audiores.2015.130	38–43	130
10.4081/audiores.2015.132	69–75	132
10.4081/audiores.2015.133	50–56	133
10.4081/audiores.2015.135	57–64	135
10.4081/audiores.2015.136	80–83	136
10.4081/audiores.2015.139	76–79	139
10.4081/audiores.2016.137	1–5	137
10.4081/audiores.2016.140	11–13	140
10.4081/audiores.2016.146	6–10	146
10.4081/audiores.2016.147	14–16	147
10.4081/audiores.2016.151	17–21	151
10.4081/audiores.2016.152	44–48	152
10.4081/audiores.2016.153	22–27	153
10.4081/audiores.2016.154	28–35	154
10.4081/audiores.2016.158	40–43	158
10.4081/audiores.2016.159	49–57	159
10.4081/audiores.2016.160	58–63	160
10.4081/audiores.2017.157	1–5	157
10.4081/audiores.2017.161	15–22	161
10.4081/audiores.2017.162	6–9	162
10.4081/audiores.2016.163	36–39	163
10.4081/audiores.2017.165	23–26	165
10.4081/audiores.2017.168	10–14	168
10.4081/audiores.2017.176	42–46	176
10.4081/audiores.2017.178	31–37	178
10.4081/audiores.2017.181	38–41	181
10.4081/audiores.2017.182	61–66	182
10.4081/audiores.2017.185	51–55	185
10.4081/audiores.2017.187	47–50	187
10.4081/audiores.2017.189	56–60	189
10.4081/audiores.2017.190	67–70	190
10.4081/audiores.2018.194	1–4	194
10.4081/audiores.2018.198	9–12	198
10.4081/audiores.2018.200	5–8	200
10.4081/audiores.2018.204	16–23	204
10.4081/audiores.2018.206	24–26	206
10.4081/audiores.2018.212	34–36	212
10.4081/audiores.2018.214	27–33	214
10.4081/audiores.2018.215	37–43	215
10.4081/audiores.2018.216	44–53	216
10.4081/audiores.2019.217	1–5	217
10.4081/audiores.2019.219	6–9	219
10.4081/audiores.2019.222	10–13	222
10.4081/audiores.2019.223	14–22	223
10.4081/audiores.2019.228	23–26	228
10.4081/audiores.2019.230	27–32	230
10.4081/audiores.2019.231	33–37	231
10.4081/audiores.2020.232	12–15	232
10.4081/audiores.2020.233	1–5	233
10.4081/audiores.2020.234	16–20	234
10.4081/audiores.2020.236	6–11	236
